# Methylome profiles to investigate gene regulation mediated by phase-variable DNA methyltransferases in serogroup B *Neisseria meningitidis*

**DOI:** 10.1128/mra.00164-26

**Published:** 2026-04-29

**Authors:** Alice Ascari, Luke V. Blakeway, Steven R. Bentley, Michael P. Jennings, Kate L. Seib

**Affiliations:** 1 Institute for Biomedicine and Glycomics, Griffith University, Gold Coast, Queensland, Australia; 2 Australian Genome Research Facility Ltd. 1974, St. Lucia, Brisbane, Queensland, Australia; Wellesley College, Wellesley, Massachusetts, USA

**Keywords:** *Neisseria meningitidis*, phase variation, DNA methyltransferase, Mod, PacBio HiFi methylome analysis, epigenetic regulation

## Abstract

*Neisseria meningitidis* serogroup B is a major cause of meningitis and septicemia. Here, we report PacBio HiFi single-molecule real-time (SMRT) methylomics data sets for strains MC58, B6116/77, and M0579. These strains encode the phase-variable DNA methyltransferases ModA11, ModA12, and ModD1, respectively, which are epigenetic regulators pivotal to meningococcal pathogenicity.

## ANNOUNCEMENT

*Neisseria meningitidis* serogroup B is a primary cause of bacterial meningitis and septicemia (1). This species encodes phase-variable DNA methyltransferases (Mods) that switch between ON-OFF states via slip-strand mispairing within the simple sequence repeat tract of their open reading frame (2–4). Mod switching drives genome-wide changes in N^6^-adenine methylation, with each Mod recognizing a unique motif. For instance, ModA11 (MC58), ModA12 (B6116/77), and ModD1 (M0579) methylate the motifs: 5′-CGY**^m6^**AG-3′, 5′-AC**^m6^**ACC-3′, and 5′-CC**^m6^**AGC-3′, respectively (3–5). Genome-wide methylation changes epigenetically modulate bacterial gene expression (4, 6). Here, we report the release of PacBio HiFi quantitative methylome resources for *N. meningitidis* strains MC58 (ModA11), B6116/77 (ModA12), and M0579 (ModD1), each profiled in a Mod ON state and an isogenic *mod::kan* knockout (KO) (Table 1).

**TABLE 1:** Per-sample sequencing data summary for PacBio HiFi methylome data sets^*a*^

Sample	Reference assembly	Genome		Annotated genes (n)	HiFi reads (n)	Yield (Gbp)	Mean length (bp)	Mean QV	Mean coverage (×)	Known m6A motifs^*b*^ (5’ to 3’)	SRA accession IDs	BioSample accession IDs
		Size (Mb)	GC (%)									
MC58 *modA11* KO	GCF_000008805.1 (ASM880v1)	2.27	51.53	2,266	1,496,415	14.4	9,601	39.1	6,221.5	CGY**A**G (ModA11) (REBASE = M.NmeMC58I) CC**A**CC (ModB1) (REBASE = M.Nme6455)	SRR36882326	SAMN54725949
MC58 *modA11* ON	GCF_000008805.1 (ASM880v1)	2.27	51.53	2,266	1,498,831	15.2	10,164	39	6,601.2	CGY**A**G (ModA11) (REBASE = M.NmeMC58I) CC**A**CC (ModB1) (REBASE = Nme6455)	SRR36882325	SAMN54725948
B6116/77 *modA12* KO	GCF_001029815.1 (ASM102981v1)	2.19	51.66	2,189	1,613,754	15.9	9,865	39	7,249.0	AC**A**CC (ModA12) (REBASE = M.Nme77I) CGA**A**T (ModB2) (REBASE = M.Nme77ORF1599P)	SRR36882330	SAMN54725945
B6116/77 *modA12* ON	GCF_001029815.1 (ASM102981v1)	2.19	51.66	2,189	1,561,565	14.9	9,553	39.1	6,800.8	AC**A**CC (ModA12) (REBASE = M.Nme77I) CGA**A**T (ModB2) (REBASE = M.Nme77ORF1599P)	SRR36882329	SAMN54725944
M0579 *modD1* KO	GCF_001029835.1 (ASM102983v1)	2.32	51.37	2,325	2,137,338	16.0	7,490	39.2	6,485.4	AC**A**CC (ModA12) (REBASE = M.Nme579II) CC**A**CC (ModB1) (REBASE = M.Nme579ORF2116P) CC**A**GC (ModD1) (REBASE = M.Nme579I)	SRR36882328	SAMN54725947
M0579 *modD1* ON	GCF_001029835.1 (ASM102983v1)	2.32	51.37	2,325	1,501,346	11.5	7,628	39.2	4,672.8	AC**A**CC (ModA12) (REBASE = M.Nme579II) CC**A**CC (ModB1) (REBASE = M.Nme579ORF2116P) CC**A**GC (ModD1) (REBASE = M.Nme579I)	SRR36882327	SAMN54725946

^*a*^ KO, knockout; ON, methyltransferase expressed; QV, quality value; m6A, N^6^-adenine methylation; REBASE, The Restriction Enzyme Database (7); Y = C or T.

^*b*^ The N^6^-adenine methylation motifs (m6A, shown in bold) of the phase-variable type III DNA methyltransferases (Mods) present in *Neisseria meningitidis* B strains MC58, B6116/77, and M0579 used in this study. Previous studies have experimentally defined these motifs for ModA11, ModA12, ModD1 (5), ModB1 (8), and ModB2 (9). The Restriction Enzyme Database assigned nomenclature is denoted for each enzyme (7).

Strains were cultured from frozen stocks in GC glycerol broth supplemented with 30 µM desferal (Sigma-Aldrich) to mimic biological iron-deplete conditions at 37°C with 200 rpm aeration. Genomic DNA was extracted from mid-logarithmic phase cells (Gel-Elute Bacterial Genomic DNA Kit; Sigma), as per manufacturer’s instructions. DNA quality and purity were assessed using pulsed-field gel electrophoresis (Femto-Pulse system) and via the CLARIOstar Plus platform, respectively.

High molecular-weight DNA was sheared (~7–10 kb) (FastPrep 96), converted to SMRTbell libraries (SMRTbell prep kit 3.0), size-selected (AmpurePB beads, >5 kb) and sequenced on the PacBio Revio system (30 h movie). HiFi data sets (~9.0-kb mean read length) yielding 87.9 Gb of high-quality sequencing data were analyzed against RefSeq assemblies, with MC58, B6116/77, and M0579 mapped to references GCF_000008805.1 (ASM880v1), GCF_001029815.1 (ASM102981v1), and GCF_001029835.1 (ASM102983v1), respectively. Alignments were generated with pbmm2 v1.12.0 (HiFi preset), with summary metrics from samtools v1.18.

PacBio HiFi SMRT sequencing enabled genome-wide methylation profiling. Modified-base information was extracted using a Fiber-seq-based workflow repurposed for endogenous prokaryotic methylation detection, applying the fire step and generating pileups while excluding non-primary and supplementary alignments. Motif methylation was quantified by locating motif instances in each reference genome (seqkit-locate v2.7.0), intersecting motif coordinates with pileup data (bedtools-intersect v2.27.1), and exporting per-motif count tables and bedGraph tracks reporting the percentage of reads modified at the target base. Motifs analysed included ModA11, ModA12, and ModD1, in addition to ModB1 (5′-CCACC-3′) (8) and ModB2 (5′-CGAAT-3′) (9). Genome-wide coverage was computed with mosdepth v0.3.10 and collated with MultiQC v1.25. The number of motif sites detected was consistent between ON and KO methylomes, with the following count per strain: MC58, 7,956 (ModA11) and 3,726 (ModB1); B6116/77, 3,969 (ModA12) and 4,349 (ModB2); and M0579, 4,281 (ModA12), 3,830 (ModB1), and 5,043 (ModD1). *De novo* motif discovery was performed using PacBio SMRT Link v25.2.0: strand-specific kinetics were generated (CCS reads; ccs-kinetics-bystrandify), reads were aligned to RefSeq assemblies (pbmm2 v1.12.0; sorted/indexed), and modified-base sites and enriched motifs (^m6^A/^m4^C) were identified using ipdSummary and pbmotifmaker (minimum motif score threshold 20).

Across all genetic backgrounds, SMRT methylomics produced high-coverage, motif-resolved methylation profiles that enable direct comparison of motif occupancy and methylation signal between Mod ON and KO conditions, with *de novo* motif discovery supporting known methyltransferase-associated motifs and identifying additional candidate motifs for further investigation.

## Data Availability

In this study, we used laboratory stocks of MC58 (cerebrospinal fluid isolate, UK, 1983), B6116/77 (Iceland, 1977), and M0579 (USA, 1993), all of which originate from patients with invasive meningococcal disease, and whose genomes and detailed metadata are publically available from pubMLST (10). Raw PacBio Revio HiFi reads (unaligned BAM files) for *N. meningitidis* serogroup B strains MC58, B6116/77, and M0579 (*mod* ON and *mod::kan*) have been deposited to the NCBI Sequence Read Archive (SRA) under BioProject PRJNA1404856. These data sets comprise raw reads; assembled transcripts were not submitted. SRA accession IDs and associated BioSample accession IDs are summarized in Table 1.

## References

[1] Pace D, Pollard AJ. 2012. Meningococcal disease: clinical presentation and sequelae. Vaccine (Auckl) 30 Suppl 2:B3–9. doi:10.1016/j.vaccine.2011.12.062 22607896

[2] Moxon R, Bayliss C, Hood D. 2006. Bacterial contingency loci: the role of simple sequence DNA repeats in bacterial adaptation. Annu Rev Genet 40:307–333. doi:10.1146/annurev.genet.40.110405.090442 17094739

[3] Seib KL, Pigozzi E, Muzzi A, Gawthorne JA, Delany I, Jennings MP, Rappuoli R. 2011. A novel epigenetic regulator associated with the hypervirulent Neisseria meningitidis clonal complex 41/44. FASEB J 25:3622–3633. doi:10.1096/fj.11-183590 21680891

[4] Srikhanta YN, Dowideit SJ, Edwards JL, Falsetta ML, Wu H-J, Harrison OB, Fox KL, Seib KL, Maguire TL, Wang AH-J, Maiden MC, Grimmond SM, Apicella MA, Jennings MP. 2009. Phasevarions mediate random switching of gene expression in pathogenic Neisseria. PLOS Pathog 5:e1000400. doi:10.1371/journal.ppat.1000400 19390608 PMC2667262

[5] Seib KL, Jen FE-C, Tan A, Scott AL, Kumar R, Power PM, Chen L-T, Wu H-J, Wang AH-J, Hill DMC, Luyten YA, Morgan RD, Roberts RJ, Maiden MCJ, Boitano M, Clark TA, Korlach J, Rao DN, Jennings MP. 2015. Specificity of the ModA11, ModA12 and ModD1 epigenetic regulator N(6)-adenine DNA methyltransferases of Neisseria meningitidis. Nucleic Acids Res 43:4150–4162. doi:10.1093/nar/gkv219 25845594 PMC4417156

[6] Srikhanta YN, Maguire TL, Stacey KJ, Grimmond SM, Jennings MP. 2005. The phasevarion: A genetic system controlling coordinated, random switching of expression of multiple genes. Proc Natl Acad Sci USA 102:5547–5551. doi:10.1073/pnas.0501169102 15802471 PMC556257

[7] Roberts RJ, Vincze T, Posfai J, Macelis D. 2023. REBASE: a database for DNA restriction and modification: enzymes, genes and genomes. Nucleic Acids Res 51:D629–D630. doi:10.1093/nar/gkac975 36318248 PMC9825431

[8] Adamczyk-Poplawska M, Lower M, Piekarowicz A. 2009. Characterization of the NgoAXP: phase-variable type III restriction-modification system in Neisseria gonorrhoeae. FEMS Microbiol Lett 300:25–35. doi:10.1111/j.1574-6968.2009.01760.x 19758331

[9] Stenmark B, Eriksson L, Thulin Hedberg S, Anton BP, Fomenkov A, Roberts RJ, Mölling P. 2021. Genome-wide methylome analysis of two strains belonging to the hypervirulent Neisseria meningitidis serogroup W ST-11 clonal complex. Sci Rep 11:6239. doi:10.1038/s41598-021-85266-7 33737546 PMC7973814

[10] Jolley KA, Bray JE, Maiden MCJ. 2018. Open-access bacterial population genomics: BIGSdb software, the PubMLST.org website and their applications. Wellcome Open Res 3:124. doi:10.12688/wellcomeopenres.14826.1 30345391 PMC6192448

